# Posterior Reversible Encephalopathy Syndrome in Acute COVID-19 Pneumonia

**DOI:** 10.1017/cjn.2021.221

**Published:** 2021-09-16

**Authors:** Laura Donaldson, Edward Margolin

**Affiliations:** Department of Ophthalmology and Vision Sciences, University of Toronto, Toronto, ON, Canada; Department of Medicine, Division of Neurology, University of Toronto, Toronto, ON, Canada

**Keywords:** COVID-19, Posterior reversible encephalopathy syndrome, Bilateral severe visual loss

A 66-year-old woman presented to the Emergency Department with shortness of breath and was found to be febrile and tachycardic. Chest CT showed bilateral ground-glass opacities, and she was admitted with a diagnosis of COVID-19 pneumonia. PCR testing confirmed that she was positive for the SARS-CoV-2 virus. Medical co-morbidities included asthma, type 2 diabetes, hypertension, and dyslipidemia. Shortly after admission, she was intubated and transferred to the ICU, where she remained for 40 days. There were no sustained episodes of hypertension. Bacterial superinfection led to Pseudomonas pneumonia, and her course was further complicated by subclavian deep vein thrombosis.

As she began to regain consciousness, she was confused and disoriented with headache, complaining that she could not see. Family members found that she did not recognize them when they entered the room, only when she could hear their voices. Two non-contrast CT scans of the brain were performed and were both interpreted as normal. When vision blurring persisted, MRI brain with diffusion-weighted imaging (DWI) was performed and showed extensive bilateral areas of T2/FLAIR hyperintensity (Figure 1), consistent with vasogenic edema and posterior reversible encephalopathy syndrome (PRES). Over the ensuing weeks, her vision recovered completely.

**Figure 1:**
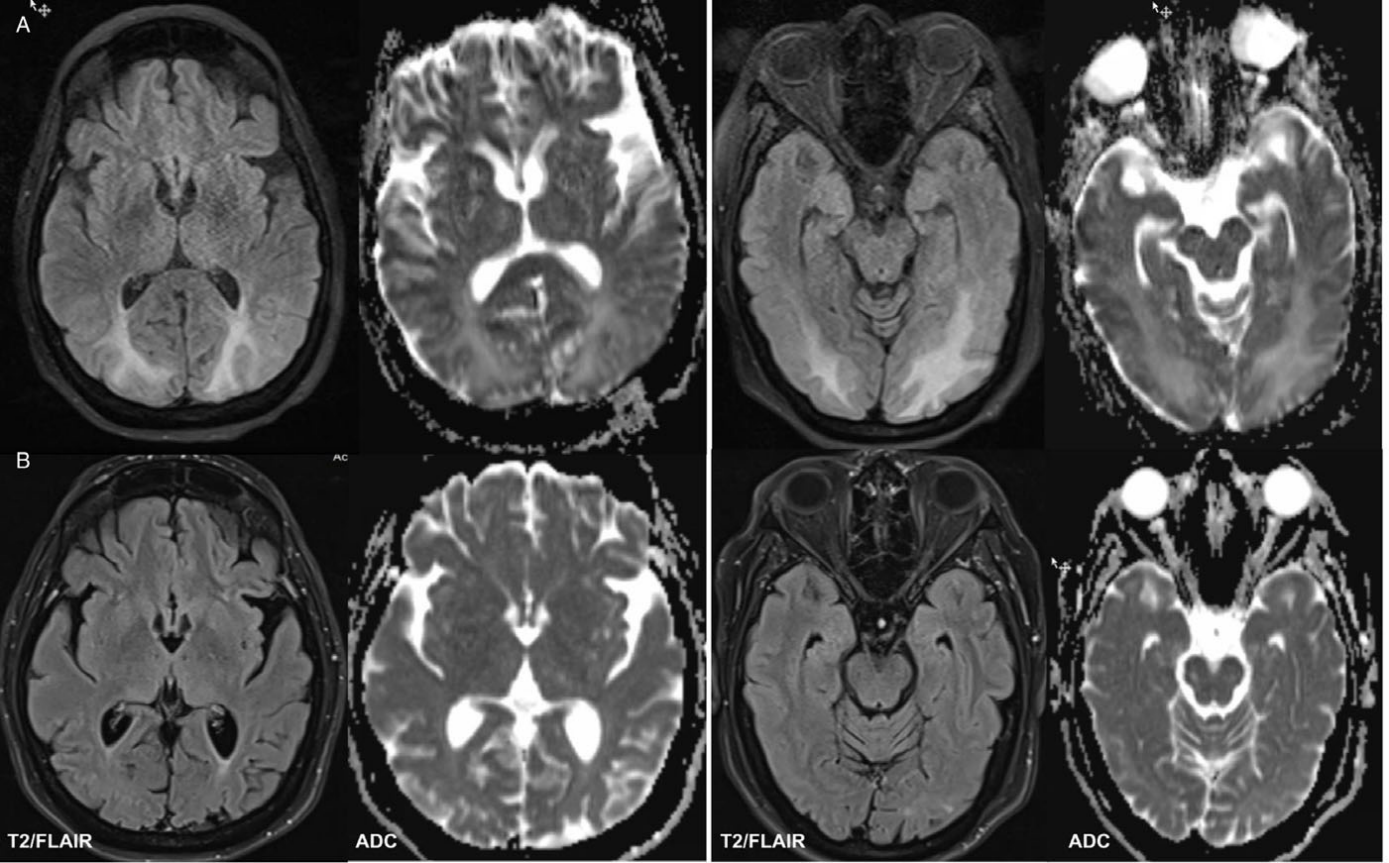
Posterior reversible encephalopathy syndrome in COVID-19. (A) Axial MRI images showing extensive bilateral regions of increased signal on T2/FLAIR sequences (left panels parieto-occipital, right panels occipital) and high signal on apparent diffusion coefficient sequences (ADC) consistent with extensive vasogenic edema. Most regions showed T2 shine through on diffusion-weighted imaging with sparse patches of restricted diffusion. (B) Follow-up MRI images showing resolution of edema.

Acutely ill patients with COVID-19 are at risk for neurologic complications including stroke, viral meningitis/encephalitis, cerebral venous thrombosis, hypoxic encephalopathy, and demyelinating disease.^1^ PRES must be included in the differential diagnosis,^2^ and appropriate neuroimaging in the form of MRI with DWI performed.^3^ PRES is a result of an incompletely understood process of failed endothelial regulation resulting in cerebral vasogenic edema and was initially described in patients with immunosuppressive drugs, renal insufficiency, or severe hypertension.^4^ Occipital and parietal lobes are most frequently affected, and symptoms include headache, altered sensorium, seizure, and vision loss. Prompt treatment to reverse the causative stimulus results in recovery of function in most cases.^5^

In COVID-19, the mechanism of PRES is most likely related to the viral-induced cytokine storm. One likely effector is tumor necrosis factor-α (TNFα), a major inflammatory cytokine released primarily by macrophages but also by many other cell types to incite multiple downstream signaling cascades and induce effects such as increased vascular permeability.^2^ Other cytokines such as interleukin-1 likely play a role and upregulate cell adhesion molecules including intercellular adhesion molecule-1 (ICAM1) and vascular cell adhesion protein-1 (VCAM1).^6,7^ An alternative hypothesis for the mechanism of PRES in these patients is dysregulation of the renin-angiotensin system (RAS) as SARS-CoV-2 spike protein binds to the angiotensin-converting enzyme-2 (ACE2) receptor to initiate cell entry.^8^ This leads to ACE2 downregulation and downstream effects on the RAS, and bradykinin–kallikrein pathway may cause endothelial dysfunction and increased vascular permeability.^9^ Drugs used to treat COVID-19 including hydroxychloroquine and tocilizumab may also be associated with PRES,^10,11^ though not a factor in this case.

PRES must be considered in COVID-19 patients with neurologic symptoms, particularly with severe bilateral vision loss. MRI of the brain with DWI is recommended in these cases to arrive at the correct diagnosis.

## References

[1] Correia AO , Feitosa PWG , Moreira JLS , et al. Neurological manifestations of COVID-19 and other coronaviruses: a systematic review. Neurol Psychiatr Brain Res. 2020;37:27–32. DOI 10.1016/j.npbr.2020.05.008. PMC7261450 32834527

[2] Parauda SC , Gao V , Gewirtz AN , et al. Posterior reversible encephalopathy syndrome in patients with COVID-19. J Neurol Sci. 2020;416:117019. DOI 10.1016/j.jns.2020.117019. 32679347 PMC7347314

[3] Pilato F , Distefano M , Calandrelli R. Posterior reversible encephalopathy syndrome and reversible cerebral vasoconstriction syndrome: clinical and radiological considerations. Front Neurol. 2020;11:494. DOI 10.3389/fneur.2020.00034. 32117007 PMC7033494

[4] Hinchey J , Chaves C , Appignani B , et al. A reversible posterior leukoencephalopathy syndrome. New Engl J Med. 1996;334:494–500. 8559202 10.1056/NEJM199602223340803

[5] Fugate JE , Rabinstein AA. Posterior reversible encephalopathy syndrome: clinical and radiological manifestations, pathophysiology, and outstanding questions. Lancet Neurol. 2015;14:914–25. DOI 10.1016/S1474-4422(15)00111-8. 26184985

[6] Racchiusa S , Mormina E , Ax A , et al. Posterior reversible encephalopathy syndrome (PRES) and infection: a systematic review of the literature. Neurol Sci. 2019;40:915–22. DOI 10.1007/s10072-018-3651-4. 30604335

[7] Gao B , Lyu C , Lerner A , et al. Controversy of posterior reversible encephalopathy syndrome: What have we learnt in the last 20 years? J Neurol Neurosurg Psychiatr. 2018;89:14–20. DOI 10.1136/jnnp-2017-316225. 28794149

[8] Hoffmann M , Kleine-Weber H , Schroeder S , et al. SARS-CoV-2 cell entry depends on ACE2 and TMPRSS2 and is blocked by a clinically proven protease inhibitor. Cell. 2020;181:271–80.e8. DOI 10.1016/j.cell.2020.02.052. 32142651 PMC7102627

[9] Bernard I , Limonta D , Mahal LK , et al. Endothelium infection and dysregulation by sars-cov-2: evidence and caveats in covid-19. Viruses. 2021;13:29. DOI 10.3390/v13010029. PMC7823949 33375371

[10] Rosa Júnior M , Borges ÉI , Fonseca APA , et al. Posterior reversible encephalopathy syndrome during treatment with tocilizumab in juvenile idiopathic arthritis. Arq Neuropsiquiatr. 2018;76:720–1. DOI 10.1590/0004-282x20180093. 30427514

[11] Talluri K , Lall N , Moreno MA , et al. Posterior reversible encephalopathy syndrome in a patient with SARS-CoV-2 infection treated with tocilizumab. Cureus. 2021;13:e13475. DOI 10.7759/cureus.13475. 33777563 PMC7987818

